# The effect of subjective socioeconomic status on wellbeing among urban older adults with intergenerational parenting: the mediating role of perceived social support

**DOI:** 10.3389/fpubh.2025.1672391

**Published:** 2025-11-03

**Authors:** Xi Luo, Ping Zhang, Fan Xu, Jing Wang, ShaoJu Xie

**Affiliations:** Department of Oncology, Deyang People’s Hospital, Deyang, Sichuan, China

**Keywords:** intergenerational parenting, subjective socioeconomic status, satisfaction with life, perceived social support, mediation modeling

## Abstract

**Objective:**

This study aimed to examine the predictive role of subjective socioeconomic status (SSES) in the wellbeing of urban older adults engaged in intergenerational parenting, and to explore the mediating role of perceived social support in the relationship between SSES and satisfaction with life.

**Methods:**

A cross-sectional survey was conducted using convenience sampling to recruit urban older residents from Deyang City who were involved in intergenerational caregiving. Data were collected through a series of standardized instruments, including a general information questionnaire, the Subjective Socioeconomic Status Scale, the Satisfaction with Life Scale, and the Perceived Social Support Scale. Pearson correlation analyses were performed using SPSS 26.0 to assess the relationships among SSS, life satisfaction, and perceived social support. Structural equation modeling via AMOS 26.0 was employed to analyze the mediating effect of perceived social support on the association between SSES and life satisfaction.

**Results:**

The urban older adults with intergenerational dependence had an average SSES score of 13.52 ± 3.72, a perceived social support score of 58.43 ± 19.15, and a satisfaction with life score of 23.96 ± 5.24. Pearson correlation analysis revealed that SSES was positively correlated with satisfaction with life, and perceived social support was also positively correlated with satisfaction with life. Mediation analysis further indicated that perceived social support partially mediated the relationship between SSES and satisfaction with life, with a mediating effect of 0.086, accounting for 17.52% of the total effect.

**Conclusion:**

The subjective socioeconomic status of urban older adults with intergenerational dependence was at a moderate-to-high level, their perceived social support was moderate, and their satisfaction with life was moderately high. SSES directly influenced satisfaction with life and also had an indirect effect through perceived social support. Families and society should provide sufficient support to the urban older adults engaged in intergenerational parenting and work to enhance their subjective socioeconomic status in order to improve their overall wellbeing.

## 1 Introduction

In May 2021, the State Council of the Central Committee of the Communist Party of China (CPC) proposed further optimization of the fertility policy, implementing the three-child policy alongside supporting measures (1). However, young parents face challenges balancing career development and childcare responsibilities, leading to the substantial child-rearing burden shifting to grandparents—a phenomenon termed intergenerational parenting (2). Intergenerational parenting refers specifically to situations where grandparents (paternal or maternal) assume primary responsibility for raising one or more grandchildren. In China, intergenerational parenting is highly prevalent, with intergenerational families emerging as one of the most significant forms of diversified family structures and functions in the 21st century (3). According to data from the China Longitudinal Aging Social Survey (CLASS), as of 2014, 70% of Chinese grandparents provided various levels of grandchild care, including 52.7% of urban grandparents assisting with childcare (4). Comparatively, in the United States, nearly 10% of grandparents (approximately 7 million) co-reside with at least one grandchild, and 37% serve as primary caregivers (5, 6)—a figure that has increased by 7% since 2009 (7). In Europe, 58% of grandmothers and 49% of grandfathers regularly provide grandchild care (8, 9). Compromised psychological and physical health among caregiving grandparents reduces their quality of life and may even shorten life expectancy (10, 11). These adverse health experiences also negatively impact grandchildren’s wellbeing (12, 13). In essence, the welfare of grandparents engaged in intergenerational parenting is affected to varying degrees during grandchild-rearing.

Most intergenerational parenting abroad stems from grandparents being compelled to assume child-rearing responsibilities due to teenage pregnancies, divorce, substance abuse, mental/physical illness, child abandonment, or abuse by the grandchildren’ parents (14, 15). In China, however, grandparents’ involvement is influenced by traditional cultural values such as “family prosperity through descendants” and “heavenly bliss,” alongside a perceived duty to support their children’s families (15). This constitutes a reciprocal intergenerational support system: grandparents provide grandchild-rearing services in exchange for old-age support from their adult children (16). While most Chinese grandparents engaged in intergenerational parenting provide caregiving support (non-custodial care) (2, 3, 6), few serve as legal guardians. Conversely, abroad, some grandparents act as custodial grandparents, assuming full child-rearing responsibilities under legal and governmental supervision (6, 10, 12, 13, 17, 18), while others provide non-custodial caregiving services. These distinct caregiving arrangements yield different wellbeing outcomes. Chinese grandparents frequently report adverse psychological effects from grandchild care (4, 18–21). Internationally, outcomes vary by care type: custodial grandparents often experience health threats, anxiety, depression, and reduced life satisfaction due to social deprivation (7, 8, 10, 14, 21); non-custodial caregiving grandparents exhibit mixed physiological and psychological outcomes. For instance, caregiving grandparents in Europe, South Korea, and the U. S. may experience cognitive improvements and health benefits through passive social participation or physical activities during childcare (17, 20, 22, 23). However, not all non-custodial grandparents benefit—some report anxiety, depression, and health deterioration (8, 12, 20). These differential outcomes are influenced not only by custody type but also by grandparents’ socioeconomic status and perceived social support (8, 23).

In summary, the life satisfaction of grandparents engaged in intergenerational parenting is affected to varying degrees during grandchild care. Research indicates that social support alleviates grandparents’ depressive symptoms and improves health outcomes (23), while higher economic wellbeing enhances their satisfaction with life and psychological-physical wellness (5, 6, 19).

This study operationalizes wellbeing through the Satisfaction with Life Scale (24). Subjective socioeconomic status (SSES) denotes an individual’s self-perceived position within the socioeconomic hierarchy. SSES correlates with mental health, satisfaction with life, health behaviors, and diverse health outcomes. Crucially, even among groups with identical economic standing, perceptual differences persist in self-assessments. SSES thus represents a more accurate evaluation metric for personal socioeconomic perception than objective measures alone (25). Perceived social support constitutes a psychometric construct assessing an individual’s subjective appraisal of received social backing. This variable serves as a core self-evaluation metric, reflecting the emotional experience of feeling supported and understood (26). Empirical evidence confirms SSES’s significant association with wellbeing (27, 28), with both SSES and perceived social support serving as positive predictors of satisfaction with life. Furthermore, higher SSES correlates strongly with elevated levels of perceived social support (29, 30).

Social Cognitive Theory—a cognition-centered theoretical model pioneered by Albert Bandura—emphasizes triadic reciprocal causation between personal factors, behavior, and environmental influences. Individual behavior is shaped not only by external environments but also regulated through internal cognitive processes. This interaction forms a “triadic reciprocity” system wherein environmental factors, individual behavior, and cognition maintain bidirectional causal relationships. The theory also underscores human agency, with its core feature being individuals’ capacity for developmental adaptation and self-renewal amidst societal evolution (31). According to Social Cognitive Theory, individuals are not merely passive products of their environment but exercise agency through cognitive processes that enable them to both adapt to and actively shape their surroundings. In this study, subjective socioeconomic status represents a cognitive factor, seeking social support constitutes a behavioral factor, and life satisfaction reflects successful environmental adaptation and improvement. Grandparents involved in intergenerational caregiving often encounter negative experiences that may diminish their wellbeing. However, enhancing their subjective socioeconomic status can empower them to seek social support more proactively. This behavioral initiative helps ameliorate their living environment, thereby promoting higher life satisfaction and ultimately enhancing overall wellbeing. Within this framework, we propose the research hypothesis that perceived social support mediates the relationship between SSES and satisfaction with life—specifically, that subjective socioeconomic status contributes to satisfaction with life through pathways influenced by perceived social support.

Current research on intergenerationally involved older adults predominantly examines cognitive capacity, health status, and negative psychological experiences (12). By contrast, minimal research has investigated the subjective socioeconomic status, perceived social support, and satisfaction with life of urban grandparents engaged in intergenerational parenting. This study therefore examines the effects of SSES and perceived social support on satisfaction with life, while investigating perceived social support’s mediating role between SSES and satisfaction with life. The findings aim to establish a theoretical foundation for enhancing wellbeing among urban grandparents in intergenerational caregiving roles.

## 2 Objects and methods

### 2.1 Subjects

Older individuals providing grandchild care in Deyang City’s urban area were selected as study subjects using convenience sampling from January to December 2023. In this study, participant recruitment was conducted through community-based sampling in selected urban districts of Deyang City. Following formal approval from community administrative authorities, the research team implemented a systematic screening process to identify eligible older adults based on predefined inclusion and exclusion criteria. Inclusion criteria: participants were grandparents aged 60 years or older who provided daily caregiving for at least 2 h to one or more grandchildren; cognitively intact with basic comprehension and cooperation abilities; provided informed consent and volunteered to participate; Grandchildren aged 0–12 years. Exclusion criteria: speech or hearing impairments; severe medical conditions (e.g., heart failure, malignant tumors, major trauma).

Calculated using the cross-sectional survey formula *n* = (t_*α*/2_s/*δ*)^2^, where α = 0.05, t_α/2_ was taken as 1.96, the permissible error δ = 0.4, and standard deviation s = 2.66 [based on prior studies (32)]. The initial sample size was 170. Accounting for a 20% attrition rate, the required sample size was 204. This study distributed 204 questionnaires and recovered 189 valid responses (effective response rate: 92.65%). The study protocol was approved by the Ethics Committee of Deyang People’s Hospital (2022–04-023-K01).

### 2.2 Survey instruments

General Information Survey Self-administered questionnaire collecting: age, gender, education level, monthly disposable income, physical health status, spouse status, number of children, and number of grandchildren raised.

Subjective Socioeconomic Status Scale Adapted from Goodman et al. (25), this 2-item scale measures community status and social status separately using a 10-point ordinal scale (1–10 per item; total score range: 2–20). Higher scores indicate higher socioeconomic status (30). The validity of this instrument is well-demonstrated across multiple studies conducted with older adults (33). Cronbach’s *α* = 0.74 originally; Cronbach’s α = 0.881 in this study.

Perceived Social Support Scale Developed by Zimet et al. (26), and translated by Jiang Qianjin, this 12-item scale covers three dimensions: family support, friend support, and other support. It uses a 7-point Likert scale (total score: 12–84), with higher scores indicating greater perceived social support. Score ranges: 12–36 (low support), 37–60 (intermediate support), 61–84 (high support) (34). The validity of this instrument is well-demonstrated across multiple studies conducted with older adults (35). Original Cronbach’s *α* = 0.90; Cronbach’s α = 0.966 in this study.

Satisfaction with Life Scale Based on Diener et al. [cited by Du et al. (24)], this 5-item scale uses a 7-point Likert scale to evaluate wellbeing. Higher scores indicate greater satisfaction with life, categorized as: 31–35 are extremely satisfied; 26–30 are satisfied; 21–25 are slightly satisfied; 20 are neutral; 15–19 are slightly dissatisfied; 10–14 as dissatisfied (36); and 5–9 as extremely dissatisfied. The validity of this instrument is well-demonstrated across multiple studies conducted with older adults (37). Original Cronbach’s *α* = 0.77; Cronbach’s α = 0.875 in this study.

### 2.3 Data collection method

This study employed a questionnaire survey method. Three uniformly trained investigators administered the questionnaires. Data collection was carried out using both paper-based questionnaires and the Wenjuanxing online survey platform. Paper questionnaires were administered to older adults who were unable to operate smartphones or were not literate. In contrast, the Wenjuanxing software^1^ was utilized for participants who could independently use smart devices. Prior to survey administration, investigators explained the purpose, significance, and completion instructions to participating intergenerational older caregivers. Questionnaires were distributed anonymously after obtaining informed consent. For older participants with limited literacy or advanced age who experienced difficulty completing the questionnaire, investigators conducted face-to-face interviews, verbally administering each item and recording responses based on participants’ oral statements. Completed questionnaires were collected and reviewed for omissions or errors. Of 204 questionnaires distributed, 189 valid questionnaires were recovered after excluding incomplete responses, incorrectly completed forms, or inconsistent answers to homogeneous items, yielding a 92.65% response rate.

### 2.4 Statistical analysis

Data analysis was performed using SPSS 26.0. Normal distribution was verified using Kolmogorov–Smirnov (K-S) tests and histogram analysis. Measurement data were presented as mean ± standard deviation, while count data were reported as frequencies and percentages. Pearson correlation analysis examined relationships between subjective socioeconomic status, perceived social support, and satisfaction with life. Common method bias was assessed using Harman’s single-factor test. Structural equation modeling was conducted with AMOS 26.0 to analyze influence pathways. The bootstrap method with bias-corrected percentile estimation tested mediation effect significance. The significance level was set at *α* = 0.05.

## 3 Results

### 3.1 General information

Among 189 urban intergenerational older caregivers, 126 (66.67%) were female and 63 (33.33%) male. Age distribution: 154 (81.5%) aged 60–65 years, 27 (14.3%) aged 66–70 years, and 8 (4.2%) aged ≥70 years. Education levels: primary school (31.22%) and junior high school (43.38%) predominated. Monthly disposable income: 27 (14.29%) < 3,000 yuan; 71 (37.57%) 3,000–5,000 yuan; 57 (30.16%) 5,000–10,000 yuan; 25 (13.22%) 10,000–15,000 yuan; 9 (4.76%) > 15,000 yuan. Health status: 91 (48.15%) subhealthy; 80 (42.33%) healthy; 18 (9.52%) with diagnosed chronic diseases. Most participants had cohabiting spouses (167, 88.36%) and one child (167, 88.36%). Number of grandchildren raised: the majority of the older adults raised one grandchild (166, 87.83%), and a small number raised two grandchildren (23, 12.17%).

### 3.2 Univariate analysis of satisfaction with life among urban older adults with intergenerational parenting

In this study, age was identified as a significant predictor of life satisfaction among caregiving grandparents (*p* < 0.05), with older age correlating with diminished life satisfaction. In contrast, other factors including gender, educational level, monthly disposable income, health status, marital status, number of children, and number of grandchildren raised showed no statistically significant associations with life satisfaction (Table 1).

**Table 1:** Univariate analysis of satisfaction with life among urban older adults with intergenerational parenting.

Dimension		n/%	Score (mean ± SD)	t/F	*P*
Gender	Male	63 (33.33)	24.92 ± 4.19	−1.969*	0.051
	Female	126 (66.67)	23.48 ± 5.65		
Age, years	60–65	154 (81.48)	24.27 ± 5.10	4.584	0.011
	66–70	27 (14.29)	23.81 ± 4.67		
	>70	8 (4.23)	18.63 ± 7.37		
Educational level	Illiterate	8 (4.23)	22.50 ± 7.11	0.348	0.845
	Primary school	59 (31.22)	23.59 ± 5.98		
	Junior high school	82 (43.38)	24.13 ± 5.16		
	High/ secondary school	35 (18.52)	24.37 ± 3.90		
	College or above	5 (2.65)	25.00 ± 2.55		
Monthly disposable income, CNY	0–2,999	27 (14.29)	24.63 ± 4.98	1.364	0.248
	3,000–4,999	71 (37.57)	24.61 ± 5.00		
	5,000–9,999	57 (30.16)	23.89 ± 5.40		
	10,000–14,999	25 (13.22)	22.36 ± 5.25		
	≥15,000	9 (4.76)	21.78 ± 6.38		
Health status	Chronic diseases diagnosed	18 (9.52)	23.39 ± 7.83	0.191	0.826
	Sub-healthy	91 (48.15)	23.88 ± 5.09		
	Healthy	80 (42.33)	24.19 ± 4.74		
Marital status	Cohabiting spouses	167 (88.36)	23.84 ± 5.15	0.527	0.592
	Divorced	10 (5.29)	24.30 ± 4.90		
	Widowed	12 (6.35)	25.42 ± 6.86		
Number of children	1	167 (88.36)	24.15 ± 5.04	1.330	0.267
	2	18 (9.53)	22.94 ± 6.10		
	≥3	4 (2.11)	20.50 ± 91.5		
Number of grandchildren raised	1	166(87.83)	24.09 ± 5.17	0.897*	0.371
	2	23(12.17)	23.04 ± 5.75		

*Indicates independent-samples *t*-test.

### 3.3 Scores of subjective socioeconomic status, perceived social support, and satisfaction with life

Urban intergenerational older caregivers demonstrated the following mean scores: subjective socioeconomic status (13.52 ± 3.72), perceived social support (58.43 ± 19.15), and satisfaction with life (23.96 ± 5.24). Dimension scores and item averages are presented in Table 2.

**Table 2:** Scores on subjective socioeconomic status, perceived social support and satisfaction with life of urban intergenerationally parenting older adults (*n* = 189, mean ± standard deviation, points).

Dimension	Number of entries	Theoretical score range	Score	Mean score of entries
Subjective socio-economic status	2	2–20	13.52 ± 3.72	6.76 ± 1.86
Perceived Social Support	12	12–84	58.43 ± 19.15	4.87 ± 1.59
Satisfaction with Life	5	6–34	23.96 ± 5.24	4.79 ± 1.05

### 3.4 Correlation analysis of subjective socioeconomic status, perceived social support and satisfaction with life among urban intergenerational older caregivers

Pearson correlation analysis revealed significant positive correlations between subjective socioeconomic status and perceived social support (*r* = 0.340, *p* < 0.01), subjective socioeconomic status and life satisfaction (*r* = 0.439, *p* < 0.01), and perceived social support and satisfaction with life (*r* = 0.353, *p* < 0.01). See Table 3 for detailed correlation analysis results.

**Table 3:** Correlation analysis between subjective socioeconomic status, perceived social support and life satisfaction among urban older adults with intergenerational parenting.

Dimension	Subjective socio-economic status	Perceived social support	Satisfaction with life
Subjective socio-economic status	1		
Perceived Social Support	0.340**	1	
Satisfaction with Life	0.439**	0.353**	1

**p < 0.01.

### 3.5 Mediation analysis of perceived social support between subjective socioeconomic status and satisfaction with life

Common method bias was assessed using Harman’s single-factor test through exploratory factor analysis. The unrotated first factor explained 27.33% of variance, below the 40% threshold (38), indicating no significant common method bias. Nevertheless, the potential for inherent biases in self-reported data cannot be entirely excluded.

**Figure 1:**
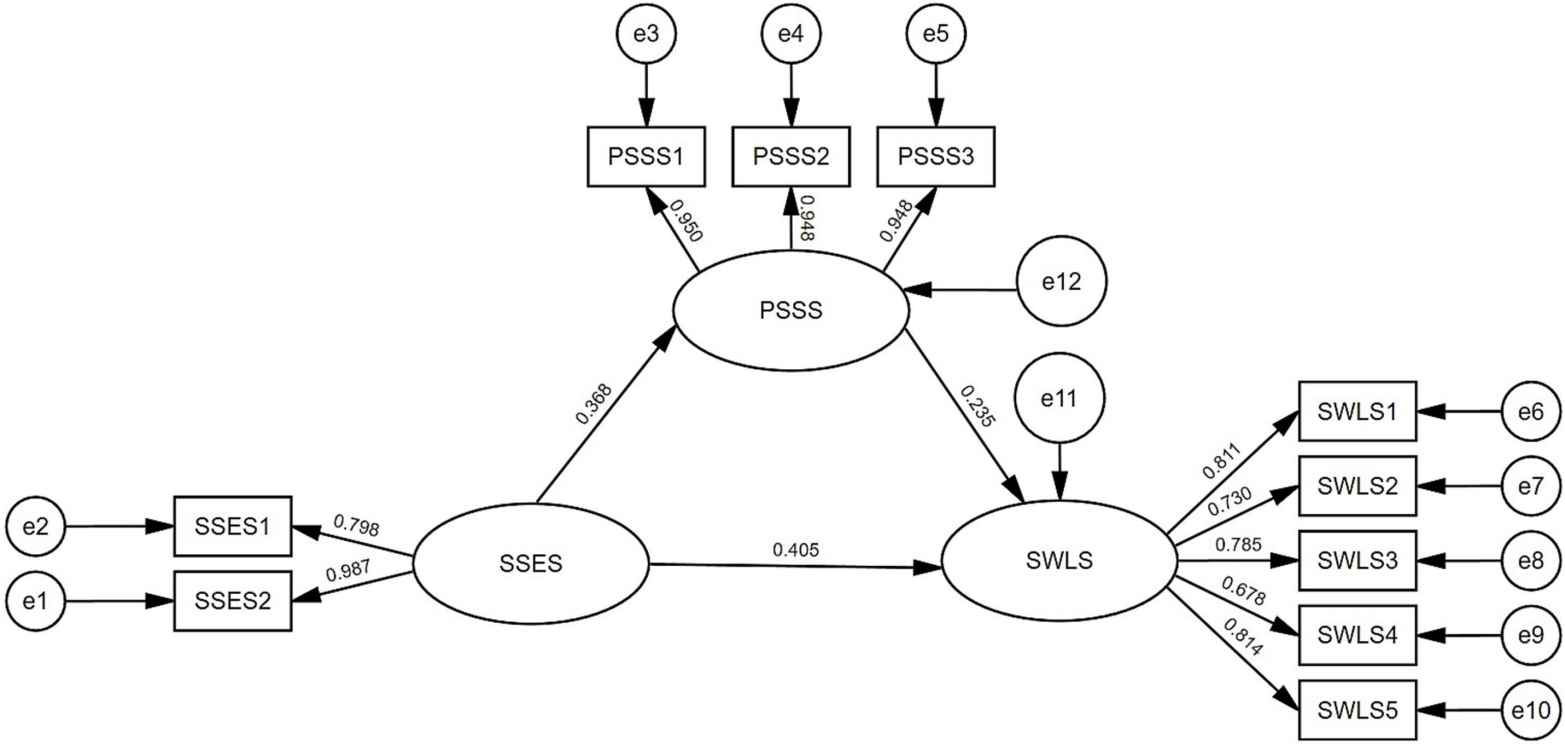
Model of the mediating effect of comprehending the impact of perceived social support on satisfaction with life through subjective socioeconomic status. SSES, denotes Subjective Socioeconomic Status; PSSS, represents Perceived Social Support Scale; SWLS, indicates Satisfaction with Life Scale.

Using AMOS 26.0, we constructed a structural equation model with: subjective socioeconomic status as the independent variable, perceived social support as the mediator variable, and Satisfaction with Life as the dependent variable, as shown in Figure 1. The model was fitted using maximum likelihood estimation, demonstrating good fit indices (39): χ^2^/df = 1.371, goodness-of-fit index GFI = 0.951, corrected fit index AGFI = 0.916, comparative fit index CFI = 0.986, Tucker Lewis index (TLI) = 0.981, value-added fit index IFI = 0.986, and root mean square of the error of approximation RMSEA = 0.056.

The mediating effect significance in the AMOS model was examined through bias-corrected bootstrap confidence interval estimation, employing 5,000 resamples with a 95% confidence interval threshold. Analysis revealed that the mediating effect of perceived social support between subjective socioeconomic status and satisfaction with life was 0.086 (95% CI: 0.024–0.176), confirming the establishment of a partial mediating effect. This mediation accounted for 17.52% of the total effect (0.086/0.491). Additionally, subjective socioeconomic status demonstrated a direct effect on satisfaction with life with a value of 0.405, constituting 82.48% of the total effect. Refer to Table 4 for comprehensive results.

**Table 4:** Mediating effects of perceived social support in the relationship between subjective socioeconomic status and satisfaction with life scale among urban older adults with intergenerational parenting.

Mediating path	Effect value	*P*	Effect share
Subjective socioeconomic status—Perceived Social Support—Satisfaction with Life Scale (indirect effect)	0.086	0.005	17.52%
Subjective socioeconomic status—Satisfaction with Life Scale (direct effect)	0.405	0.001	82.48%
Subjective socioeconomic status—Satisfaction with Life Scale (total effect)	0.491	0.000	/

## 4 Discussion

### 4.1 Univariate analysis of satisfaction with life among urban older adults with intergenerational parenting

In this study, age was identified as a significant predictor of life satisfaction among caregiving grandparents (*p* < 0.05), with older age correlating with diminished life satisfaction. These findings are consistent with those reported by Guo et al. (4) who highlighted the substantial physical and psychological demands associated with grandchild care. Older adults often experience feelings of being overwhelmed and express concern that perceived shortcomings may lead to criticism from their adult children, ultimately contributing to reduced life satisfaction. We therefore recommend that adult children take proactive steps to monitor their parents’ physical and mental wellbeing, share responsibilities related to household and child-care tasks, and encourage regular health examinations and the adoption of health-promoting behaviors. Furthermore, community-based organizations should be engaged to provide respite care services—such as temporary daycare—and facilitate intergenerational activities, allowing grandparents periodic breaks from caregiving duties. Such measures may alleviate caregiver burden and promote wellbeing. In contrast to the findings of Ju et al. (33) our analysis did not identify educational attainment, income, health status, or the number of grandchildren cared for as significant predictors of life satisfaction. Whereas Ju et al. demonstrated that higher socioeconomic status enhances wellbeing by reducing social constraints and promoting respect and instrumental support, this discrepancy may be attributable to contextual differences in sampling. In the present study, nearly all grandparents co-resided with their adult children, who served as the primary financial providers; the grandparents’ role largely involved custodial care, which may have diminished the relevance of their own economic resources. Similarly, previously observed negative effects of caring for a greater number of grandchildren particularly among those without a spouse (40) were not evident in our sample, likely because the vast majority of participants lived with a partner. Future studies would benefit from larger and more diverse samples that deliberately include grandparents with varying living arrangements, marital statuses, and socioeconomic backgrounds to improve the generalizability of the findings.

### 4.2 The subjective socioeconomic status of urban intergenerational older adults is at an upper-middle level, with moderate perceived social support and moderately high satisfaction with life

The subjective socioeconomic status score of urban intergenerational older adults in this study was 13.52 ± 3.72, indicating an upper-middle level status. This finding is consistent with previous research by Chinese scholars including Ju et al. (33). Several factors may contribute to this result. First, most urban older adults have stable pension income or personal savings that provide fundamental financial security, with some even being able to offer financial support to their children. Second, for those rural grandparents who migrate to cities to help care for grandchildren while their adult children work, they typically receive financial support through intergenerational transfers from their children. During the process of providing grandchild care, these older adults experience multiple benefits. They not only help reduce their children’s childcare burdens, but also receive gratitude and respect in return, which enhances their family status. Furthermore, through daily interactions with grandchildren, many grandparents develop close emotional bonds that strengthen their self-identity and sense of purpose. Additionally, while caring for grandchildren, some grandparents actively participate in various community activities such as peer support groups and intergenerational events, which help expand their social networks and community engagement. These findings have important implications.

Regarding perceived social support, the score in our study was 58.43 ± 19.15, indicating a moderate level of support, which aligns with findings from Zhang Yan and other Chinese researchers (40). This phenomenon can be explained from two perspectives. On the positive side, while providing grandchild care, these older adults often receive emotional support and recognition from their children. Their participation in peer communication and social activities during caregiving also enhances their sense of being supported (41). However, we must recognize the challenges. Providing full-time grandchild care is often physically and emotionally demanding. Many grandparents simultaneously undertake various household chores to help reduce their children’s burdens (42), leaving them little personal time. Even when they participate in social activities, these are usually limited to interactions with other grandparents during childcare rather than maintaining their original social circles. This situation may lead to social isolation and reduced perceived support. Moreover, the intensive caregiving responsibilities often leave them insufficient time for medical check-ups and self-care activities. These results clearly demonstrate that providing grandchild care presents both opportunities and challenges for older caregivers.

The satisfaction with life score of intergenerationally raised older participants in this study (23.96 ± 5.24) indicates a moderately satisfied state, which aligns with the findings reported by Dong et al. (16). This phenomenon may be attributed to several factors. First, the co-residence arrangement with children and grandchildren while providing childcare services likely reduces feelings of loneliness among the older adults. Second, the act of caring for grandchildren enhances the older adults’ sense of self-identity through established emotional bonds with younger generations. Third, adult children often perceive this grandparental care as emotional support, leading to increased recognition and appreciation that validates the older adults’ contributions and consequently elevates their satisfaction with life. These observations are consistent with the research conclusions of Danielsbacka et al. (8). These findings suggest that the multigenerational co-residence care model may be particularly beneficial for the wellbeing of intergenerationally raised older adults. This arrangement offers dual advantages: it increases intergenerational contact time while potentially mitigating caregiving stress. Furthermore, it is essential to foster awareness among younger family members regarding the older adults’ contributions, thereby strengthening the older generation’s self-identity and overall wellbeing. Such an approach appears conducive to promoting mutual health benefits for both the older adults and their grandchildren.

In summary, when addressing the wellbeing of intergenerationally raised older populations, government agencies, social institutions, and families should comprehensively consider both the direct and indirect effects of perceived social support on satisfaction with life. This approach should focus on improving their social support systems while simultaneously enhancing their self-cognition and social identity within community contexts. The implementation of support measures should be approached from four key directions that correspond to the core components of social support. At the family levels, efforts should be made to encourage and guide family members—including children and grandchildren—to explicitly acknowledge and appreciate the contributions of older adults. This can be achieved through practices such as organizing family appreciation events on weekends, arranging for children to temporarily relieve their parents from grandparenting duties during time off, and formally expressing gratitude to make the older adults feel valued and enhance their self-identity. Simultaneously, older adults should be encouraged to take regular breaks, engage in social activities, and maintain contact with relatives and friends. At the community level, themed activities tailored for grandparents—such as nostalgia gatherings, traditional song and dance performances, Tai Chi instruction, smartphone literacy workshops, and demonstrations of traditional handicrafts—should be actively organized. These initiatives provide respite from childcare and household chores, allowing older adults to interact with peers and enjoy age-appropriate socialization. At the governmental level, grassroots personnel should be mobilized to conduct community outreach activities that disseminate information about existing national health care mechanisms and policies for the older adults. Raising awareness of these supports can strengthen perceived social backing and thereby contribute to wellbeing. At the societal level, partnerships with social welfare organizations should be fostered to provide health clinics offering chronic disease management education, skill-building programs to help older adults adapt to contemporary caregiving roles, and intergenerational activities such as handicraft and painting sessions that grandparents can attend with their grandchildren.

### 4.3 The positive predictive relationship between subjective socioeconomic status and satisfaction with life

Pearson correlation analysis in this study revealed a significant positive correlation between subjective socioeconomic status scores and satisfaction with life among intergenerationally raised older adults (*r* = 0.439, *p* < 0.01), indicating that higher perceived socioeconomic status corresponds with greater satisfaction with life. Mediation effect analysis demonstrated that subjective socioeconomic status directly predicts satisfaction with life (*β* = 0.360, *p* < 0.01), with this direct effect accounting for 82.48% of the total effect. These results confirm that subjective socioeconomic status serves as a positive predictor of satisfaction with life in this population, consistent with findings reported by Li et al. (30) in college student populations. Subjective socioeconomic status represents an individual’s self-assessment of their social standing. When intergenerationally raised older adults perceive their socioeconomic status favorably, their enhanced satisfaction with life aligns with the traditional Chinese philosophy of “contentment brings happiness.” From the perspective of Maslow’s hierarchy of needs theory, once basic physiological and safety needs are met, individuals pursue higher-level needs for love and belonging. For these older individuals, satisfaction with socioeconomic status enables them to provide both economic and practical support to their children while alleviating caregiving burdens. This dynamic fosters reciprocal recognition and gratitude from younger generations, fulfilling the older adults’ need for love and belonging while strengthening self-identity, thereby contributing to improved satisfaction with life.

### 4.4 The mediating role of perceived social support in urban intergenerational older adults

The findings of this study demonstrate that perceived social support partially mediates the relationship between subjective socioeconomic status and satisfaction with life among urban intergenerational older adults. Specifically, perceived social support indirectly predicts satisfaction with life through subjective socioeconomic status (*β* = 0.086, *p* < 0.01), accounting for 17.52% of the total effect. This indicates that intergenerationally raised older adults who perceive higher levels of social support during grandchild caregiving are better able to recognize support from family, friends, and society. This enhanced perception subsequently elevates their subjective socioeconomic status, making them feel positioned at a higher level within their social group, thereby increasing satisfaction with life. From a theoretical perspective, social cognitive theory posits a dynamic interaction between individuals and their environment, where behavior is influenced by both external circumstances and internal cognitive processes. This theoretical framework emphasizes human capacity for self-development, adaptation to change, and continuous self-renewal (23). Given humans’ inherent social nature and the profound influence of traditional Chinese cultural values—including concepts of “family unity,” “three generations under one roof,” and “the joys of grandchildren”—intergenerational caregiving assumes particular significance (2). In contrast to the nuclear family structure prevalent in Western societies, where parents and children typically become more independent after reaching adulthood, intergenerational ties in Chinese families often remain strong throughout life. When adult children in China encounter challenges in balancing childcare and professional responsibilities, they frequently turn to their parents for support. Older adults, in turn, are generally willing to provide assistance by engaging in intergenerational childcare. This phenomenon can be attributed to several factors. First, grandparental involvement in childcare reduces the time and effort required by young parents, enabling them to devote greater energy to their careers, thereby increasing their income and professional advancement. This aligns with the fundamental aspiration of many parents to support their children’s socioeconomic development. Second, rooted in traditional Chinese cultural values, many older adults perceive caring for their grandchildren not only as a familial responsibility but also as a moral obligation. This notion of selfless dedication, often without expectation of reciprocation, reflects core principles of intergenerational solidarity and family continuity in Chinese society. Third, older caregivers often derive meaningful rewards from their roles. These include material compensation provided by their children in recognition of childcare efforts, as well as emotional fulfillment—sometimes described as “the joy of family intimacy”—that comes from spending time with their grandchildren. This culturally embedded practice reinforces a cognitive inclination among older adults to contribute to their children’s wellbeing and reduce their burdens, thereby enhancing their own subjective socioeconomic status. The process of actively seeking social support in the context of grandparenting can be viewed as a form of cognitively motivated behavioral adaptation aimed at improving a stressful caregiving environment and mitigating negative experiences, ultimately leading to enhanced wellbeing. This dynamic mirrors the “triadic reciprocal determinism” posited by social cognitive theory, which emphasizes the continuous interplay between personal cognition, behavior, and environmental factors. It also resonates with a broader Chinese cultural ethos that emphasizes proactive adaptation to and transformation of challenging circumstances. These findings show partial alignment with experiences of caregiving grandparents in other regions (e.g., Europe, South Korea, and the United States), where some report cognitive benefits and health improvements through passive social participation or physical activity during grandchild care (17, 20, 22, 23). However, notable cultural differences exist. Unlike their Chinese counterparts who emphasize family continuity and intergenerational dedication under cultural paradigms of family harmony, Western custodial grandparents often face more challenging circumstances (7, 8, 10, 14, 21). Many assume caregiving responsibilities due to adverse situations like teenage pregnancies, parental divorce, substance abuse, mental/physical illness, abandonment, or child abuse (12–14). These grandparents frequently experience significant financial pressures, governmental and community oversight, and crucially, lack the culturally embedded intergenerational support systems characteristic of Chinese society. The absence of these protective factors likely contributes to the heightened prevalence of health concerns, anxiety, depression, and diminished satisfaction with life reported among custodial grandparents in Western contexts.

## 5 Limitations of this study

We acknowledge that socioeconomic status was not employed as a stratification variable in the present sampling strategy. We highlight that future studies will utilize more rigorous designs, including stratified sampling based on socioeconomic status, to enhance sample representativeness.

This study has several limitations that should be acknowledged. First, the study sample was recruited from a single city (Deyang) using convenience sampling, which may limit the representativeness of the participants. Therefore, the findings may not fully reflect the situations of all urban older adults engaged in intergenerational parenting, particularly those residing in regions with differing socioeconomic and cultural contexts. Furthermore, this study did not include rural older adults, who may differ systematically from their urban counterparts in aspects such as socioeconomic status, family structures, and access to social support. Consequently, caution is warranted when generalizing these results. Future research should employ more representative, multi-center sampling strategies and incorporate rural populations to examine the cross-regional and cross-population applicability of the current findings, thereby enhancing the external validity of the results.

The most significant limitation of this study stems from its cross-sectional design, which inherently limits causal inference. While our findings are consistent with a model in which subjective socioeconomic status enhances wellbeing both directly and indirectly through perceived social support, alternative explanations (e.g., reverse causality) cannot be ruled out. For instance, it is possible that individuals with higher wellbeing perceive their social status more positively or are more proactive in seeking support. To unequivocally establish causality and clarify the temporal order of these variables, future research should employ longitudinal designs with multiple time points. Such designs would allow researchers to test cross-lagged relationships and better examine how changes in subjective socioeconomic status predict subsequent changes in social support and wellbeing.

## 6 Conclusion

In summary, enhancing the wellbeing of older adults engaged in intergenerational childcare requires a multilevel approach that addresses both structural and psychosocial factors. Government agencies, social institutions, and families should collaboratively strengthen perceived social support recognizing its direct and indirect effects on life satisfaction while also improving elders’ self-perception and social identity within the community. Concretely, family members should actively acknowledge and express gratitude for the contributions of older adults, reinforcing their sense of value. Simultaneously, older individuals ought to be encouraged to participate in social activities and maintain meaningful relationships to foster belonging and purpose. At the societal level, policymakers should advance older adults-care policies and develop comprehensive support networks that facilitate integration and security. Finally, families have a critical role in providing holistic old-age support—encompassing financial, practical, and emotional dimensions—thereby elevating subjective socioeconomic status and overall wellbeing.

## Data Availability

The raw data supporting the conclusions of this article will be made available by the authors, without undue reservation.

## Footnotes

^1^ https://www.wjx.cn

## References

[1] Mu G. The three-child policy and the optimization of fertility in China: background, outlook and vision. J Yangzhou Univ. (2021) 25:65–77. doi: 10.19411/j.cnki.1007-7030.2021.04.006

[2] Yu J Guo K Mai D. Intergenerational rearing culture, reproductive age choice and population fertility in China. Finan Res. (2023) 8:189–206.

[3] Song L Feng X. Intergenerational parenting: an analytical framework from the perspective of grandparents. J Shaanxi Normal Univ. (2018) 47:83–9. doi: 10.15983/j.cnki.sxss.2018.0123

[4] Guo C Chen J Wang X. Intergenerational grandparenting in Hangzhou, life satisfaction and its impact on quality of life. Chin J Gerontol. (2022) 42:2004–8. doi: 10.3969/j.issn.1005-9202.2022.08.061

[5] Sneed RS Schulz R. Grandparent caregiving, race, and cognitive functioning in a population-based sample of older adults. J Aging Health. (2019) 31:415–38. doi: 10.1177/0898264317733362, PMID: 29254404 PMC6474833

[6] Caputo J Cagney KA Waite L. Keeping us young? Grandchild caregiving and older adults' cognitive functioning. J Marriage Fam. (2024) 86:633–54. doi: 10.1111/jomf.12945, PMID: 38682083 PMC11045009

[7] McKay IT Nadorff DK. The impact of custodial grandparenting on cognitive performance in a longitudinal sample of grandparents raising grandchildren. J Fam Issues. (2021) 42:2242–62. doi: 10.1177/0192513X20976729

[8] Danielsbacka M Křenková L Tanskanen AO. Grandparenting, health, and well-being: a systematic literature review. Eur J Ageing. (2022) 19:341–68. doi: 10.1007/s10433-021-00674-y, PMID: 36052183 PMC9424377

[9] Arpino B Bordone V. Does grandparenting pay off? The effect of child care on grandparents' cognitive functioning. J Marriage Fam. (2014) 76:337–51. doi: 10.1111/jomf.12096

[10] Mendoza AN Fruhauf CA Bundy-Fazioli K Weil J. Understanding Latino grandparents raising grandchildren through a bioecological Lens. Int J Aging Hum Dev. (2018) 86:281–305. doi: 10.1177/0091415017702907, PMID: 28413885

[11] Hayslip B Kaminski PL. Grandparents raising their grandchildren: a review of the literature and suggestions for practice. Gerontologist. (2005) 45:262–9. doi: 10.1093/geront/45.2.262, PMID: 15799992

[12] Kelley SJ Whitley DM Escarra SR Zheng R Horne EM Warren GL. The mental health well-being of grandparents raising grandchildren: a systematic review and meta-analysis. Marriage Fam Rev. (2021) 57:329–45. doi: 10.1080/01494929.2020.1861163

[13] Hayslip B Fruhauf CA Dolbin-MacNab ML. Grandparents raising grandchildren: what have we learned over the past decade? Gerontologist. (2019) 59:e152–63. doi: 10.1093/geront/gnx106, PMID: 28666363

[14] Martin A Albrechtsons D MacDonald N Aumeerally N Wong T. Becoming parents again: challenges affecting grandparent primary caregivers raising their grandchildren. Paediatr Child Health. (2021) 26:e166–71. doi: 10.1093/pch/pxaa052, PMID: 34131461 PMC8194772

[15] Yu Y Gong L. Fertility policy, fertility and family old age. China's Industr Econ. (2021) 5:38–56. doi: 10.19581/j.cnki.ciejournal.2021.05.002

[16] Dong X Ling H Yang T Wang K. Grandchild care and life satisfaction of older adults: empirical evidence from China. Front Psychol. (2023) 14:1081559. doi: 10.3389/fpsyg.2023.1081559, PMID: 36814668 PMC9939526

[17] Chen F Mair CA Bao L Yang YC. Race/ethnic differentials in the health consequences of caring for grandchildren for grandparents. J Gerontol Ser B Psychol Sci Soc Sci. (2015) 70:793–803. doi: 10.1093/geronb/gbu160, PMID: 25481922 PMC4635642

[18] Xu L Tang F Li LW Dong XQ. Grandparent caregiving and psychological well-being among Chinese American older adults—the roles of caregiving burden and pressure. J Gerontol A Biol Sci Med Sci. (2017) 72:S56–62. doi: 10.1093/gerona/glw186, PMID: 28575256

[19] Zeng Y Chen Y-C Lum TYS. Longitudinal impacts of grandparent caregiving on cognitive, mental, and physical health in China. Aging Ment Health. (2021) 25:2053–60. doi: 10.1080/13607863.2020.1856779, PMID: 33291945

[20] Kim J Park EC Choi Y Lee H Lee SG. The impact of intensive grandchild care on depressive symptoms among older Koreans. Int J Geriatr Psychiatry. (2017) 32:1381–91. doi: 10.1002/gps.4625, PMID: 27905151

[21] Clottey EN Scott AJ Alfonso ML. Grandparent caregiving among rural African Americans in a community in the American south: challenges to health and wellbeing. Rural Remote Health. (2015) 15:3313. doi: 10.22605/RRH3313, PMID: 26270646

[22] Ahn T Choi KD. Grandparent caregiving and cognitive functioning among older people: evidence from Korea. Rev Econ Household. (2019) 17:553–86. doi: 10.1007/s11150-018-9413-5

[23] Bussey K Bandura A. Social cognitive theory of gender development and differentiation. Psychol Rev. (1999) 106:676–713. doi: 10.1037/0033-295X.106.4.676, PMID: 10560326

[24] Du H King RB Chi P. Income inequality is detrimental to long-term well-being: a large-scale longitudinal investigation in China. Soc Sci Med. (2019) 232:120–8. doi: 10.1016/j.socscimed.2019.04.043, PMID: 31077973

[25] Goodman E Adler NE Daniels SR Morrison JA Slap GB Dolan LM. Impact of objective and subjective social status on obesity in a biracial cohort of adolescents. Obes Res. (2003) 11:1018–26. doi: 10.1038/oby.2003.140, PMID: 12917508

[26] Zimet GD Powell SS Farley GK Werkman S Berkoff KA. Psychometric characteristics of the multidimensional scale of perceived social support. J Pers Assess. (1990) 55:610–7. doi: 10.1080/00223891.1990.9674095, PMID: 2280326

[27] Yang H Zhang H Gong S. The effect of subjective socioeconomic status on the subjective well-being of secondary school nursing students: a multiple mediation model. Chin J Clin Psychol. (2021) 29:608–13. doi: 10.16128/j.cnki.1005-3611.2021.03.033

[28] Liu X Li J Zhang L Xiang C Wu Y. The relationship between subjective socioeconomic status and psychological well-being: the mediating role of creativity and the moderating role of security. Psychol Behav Res. (2023) 21:138–44. doi: 10.12139/j.1672-0628.2023.01.020

[29] Chang B Huang J Fang J. Subjective socioeconomic status and life satisfaction of college students: chain mediation effects. Chin J Health Psychol. (2022):1–10. doi: 10.13342/j.cnki.cjhp.2021.06.033

[30] Li Q Liu F Li Z. The effect of subjective socioeconomic status on well-being in early adulthood: a chain-mediated model. Chin J Clin Psychol. (2022) 30:802–7. doi: 10.16128/j.cnki.1005-3611.2022.04.010

[31] Wang X Zhong R. A study of the mechanism of the effect of new media exposure on depression in older adults--an empirical study based on social cognitive theory. J Yunn Univ National. (2025) 42:62–71. doi: 10.13727/j.cnki.53-1191/c.20250427.005

[32] Liang T Chen H Huang Y Lin X. A study on the correlation between subjective well-being and social support of emergency department nurses in Fuzhou area. Chin J Nurs. (2015) 50:1014–7. doi: 10.3761/j.issn.0254-1769.2015.08.027

[33] Ju H Sun X Liu X. The effect of subjective socioeconomic status on depressive symptoms in older adults. Med Soc. (2022) 35:79–84. doi: 10.13723/j.yxysh.2022.04.016

[34] Sun Z Du K. The effect of navigating social support on the subjective well-being of the elderly mobile population. Soft Sci Health. (2024) 38:34–7. doi: 10.3969/j.issn.1003-2800.2024.04.008

[35] Fu L Zhang X Sun X. A study of the mediating effects of perceived stress and appreciative social support between perceived ageism and suicidal ideation in older adults in nursing facilities. J Nurs. (2023) 30:1–5. doi: 10.16460/j.issn1008-9969.2023.20.001

[36] Diener E Emmons RA Larsen RJ Griffin S. The satisfaction with life scale. J Pers Assess. (1985) 49:71–5. Epub 1985/02/01. doi: 10.1207/s15327752jpa4901_13, PMID: 16367493

[37] Sha S Li Y Liu S Tan C Zhao A Li R. Mediating effects of life satisfaction between e-health literacy and quality of life among older adults in nursing facilities. Occupat Health. (2025) 41:646–50. doi: 10.13329/j.cnki.zyyjk.2025.0057

[38] Tang D Wen Z. Common method bias testing: issues and recommendations. Psychol Sci. (2020) 43:215–23.

[39] Wang Y Wen Z Li W Fang J. Research and model development of domestic structural equation modeling methods in 20 years of the new century. Adv Psychol Sci. (2022) 30:1715–33. doi: 10.3724/SP.J.1042.2022.01715

[40] Cheng X Min S Guo R Wang R Li B. The effect of caring for grandchildren on grandparents' depressive symptoms - the mediating role of intergenerational communication. Modern Prevent Med. (2023) 50:332:1288–92. doi: 10.20043/j.cnki.MPM.202208067

[41] Zhang Y Mei D Xing F Wang F Jing L Zhang P. Correlation between perceived social support, coping styles and resilience among community-dwelling older adults. Chin J Gerontol. (2024) 44:1755–8. doi: 10.3969/j.issn.1005-9202.2024.07.052

[42] Li Y Gao J. Does intergenerational parenting affect the social participation behavior of older adults? Labor Econ Res. (2024) 12:91–116.

